# COVID-19 and Organized Crime: Strategies employed by criminal groups to increase their profits and power in the first months of the pandemic

**DOI:** 10.1007/s12117-021-09434-x

**Published:** 2021-09-13

**Authors:** Alberto Aziani, Gianluca A. Bertoni, Maria Jofre, Michele Riccardi

**Affiliations:** Università Cattolica del Sacro Cuore and Transcrime, Largo Gemelli 1, 20123 Milan, Italy

**Keywords:** Illegal Governance, Embezzlement, Infiltration, Coronavirus, Social Distancing

## Abstract

The COVID-19 pandemic has created new opportunities for organized criminal groups and confronted them with new challenges. Analysis of how these groups have reacted to the pandemic yields better understanding of how they work and enables the devising of more effective counter-strategies. To this end, we identified illustrative cases regarding the provision of illegal governance and infiltration of the legal economy by conducting a systematic content analysis of international media articles and institutional reports published during the first eight months after the outbreak of the pandemic (January to August 2020). These cases were further analyzed in order to cluster the behavior of criminal groups in response to the COVID-19 emergency, and the means by which they tried to exploit the pandemic to strengthen their political and economic power. We found that different governance-type criminal groups proposed themselves as institutions able to mitigate the burdens imposed by the pandemic by providing support to people in need and enforcing social-distancing measures. Further, identified cases did not provide evidence of groups devoted to the provision of illicit services and goods assuming any governance role. In this respect, the available evidence supports previous knowledge about organized crime. Cases of misappropriation of public funds and organized crime infiltration of the legal economy seem less common, at least in the first phase of the pandemic. The wholesale distribution of pharmaceuticals and medicines has been the sector targeted the most.

## Introduction

Since the beginning of 2020, the COVID-19 pandemic and the related containment policies have modified the environment in which organized crime groups (OCGs) operate. COVID-19 generated (a) pressure for rapid and effective governance to contain the pandemic, and (b) an economic recession. These changes may affect OCGs’ strategies, thus providing an opportunity to investigate how these organizations work when dealing with global crises. Finding evidence on how some OCGs have reacted to the social and economic transformation introduced by COVID-19 is important to devise fair and effective policies aimed both at containing the pandemic and sustaining the economic system. Moreover, it supports the fight against organized crime (OC) in a world that is in many ways different from what it used to be before COVID-19, and where official figures and reports are not always available in a timely manner. Indeed, due to the transformations induced by the pandemic, OCGs had greater opportunities to further infiltrate the legal economy and to strengthen their illegal governance, possibly posing additional threats for governments.

Analyzing OCGs’ reaction to COVID-19 helps also to gain greater insights into contemporary OCGs. Indeed, two distinctive features of structured OCGs are possibly relevant in the pandemic and post-pandemic scenarios. On the one hand, it is OCGs’ capacity to present themselves as providers of governance, which relates to the need for effective decision-making induced by the pandemic. On the other hand, the same groups have the capacity to infiltrate the legal economy, which relates to the economic crisis induced by COVID-19. The COVID-19 crisis allows investigation on OCGs that emerge as providers of governance, how they do so, and in what circumstances. Similarly, it is possible to examine evidence on industries OCGs appear to target the most and their reactions to governments’ investments to meet healthcare costs and support their economies.

OCGs’ provision of illegal governance and their infiltration of the legal economy since the onset of the pandemic are much debated by international and supranational organizations, scholars and activists. Nonetheless, empirical studies on these topics are scant. This study conducts an exploratory investigation of this issue. It does so by analyzing illustrative cases of OCGs’ activities reported by media and international organizations, at international level, covering the period from January to August 2020. In doing so, it provides a qualitative assessment based on reported cases and official evidence rather than on theoretical speculations. No academic studies of this kind are currently available, to the best of our knowledge.

The paper is structured as follows. The next section underlines the gap of knowledge that this study aims to address by providing a survey of the few available empirical publications on COVID-19 and organized crime followed by a description of the various criminal dynamics investigated by this study in the broader literature on OCGs. The methodological section describes the features of the content analysis at the basis of this study and the limitations of using media sources for data collection. The results section highlights how different types of OCGs appear to provide different forms of governance and the mechanisms behind OC infiltration of the legal economy in the aftermath of the pandemic as emerging from the analysis of identified cases. Then, in the Discussion section, we analyze both sets of results in light of the theoretical literature. The paper concludes by considering policy implications.

## Literature review

Since the onset of COVID-19 in early 2020, a number of scientists have investigated the impact of the virus on many volume and urban crimes in different parts of the world as well as in the cyber-space (e.g., Ashby 2020; Campedelli et al. 2020; Gerell et al. 2020; Lallie et al. 2021). Moreover, several studies have considered the challenges posed by the possible reactions of OCGs to the COVID-19 pandemic (e.g., Dellasega and Vorrath 2020; GITOC 2020a). These studies on OCGs mostly investigate various types of social and institutional vulnerabilities, and they outline possible future scenarios on the basis of theoretical reasoning and the observation of macro trends such as the drop in international migration or the increase in coronavirus-related phishing frauds. However, empirical studies investigating organized criminal activities are rare, as the observation of OCGs’ actions is difficult, sometime impossible, in such a short period. The specific topics investigated by the few available studies are quite various ranging from the provision of illegal governance to the evolution of OCGs’ criminal activities. In terms of geo-political focus, most studies concentrated on Latin American countries; while only an analysis considers a different area: the Western Balkans.

Bruce et al. (2020) examine how different forms of territorial control have been enforced by drug trafficking gangs and paramilitary groups during the COVID-19 epidemic in the city of Rio de Janeiro. Their results indicate that neighborhoods with a strong presence of drug gangs have had fewer casualties attributable to COVID-19 than have neighborhoods where the government has de facto control. By contrast, COVID-19-related deaths have been higher in neighborhoods controlled by paramilitary groups. Bruce et al. (2020) interpret their results in light of the fact that militias’ profits are likely more affected by restriction orders, while drug gangs’ profits depend little on the income of the population living in the areas under their control. Hence, the opportunity cost of implementing social-distance measures has been higher for militias than for drug gangs.

The provision of illegal governance is at the center also of the study by Gomez (2020), which focuses on Colombia and Mexico. Gomez (2020) reports different episodes of provision of illegal governance (e.g., distribution of food, money, medicines, face masks, organization of checkpoints) by OCGs in the periphery of Medellín and in several areas of Mexico. The study underlines how criminal groups have been more effective than the public institutions in providing solution to local communities and reflects on the severe impact that this fact may have on the legitimacy of the legal governments of the two countries.

Whether the COVID-19 pandemic has had any effect on the activities of drug-trafficking organizations in Mexico City is, instead, the research interest of Balmori de la Miyar et al. (2021) and colleagues. To proxy OCGs’ activities, they focus on kidnapping, homicides, and extortions while the study does not investigate neither illegal governance nor infiltration of the legal economy. Their event-study analysis indicates no effect on kidnapping and homicides. The rate of extortions also did not change during the first seven weeks of observation; then, it declined in weeks eight and ten. The result then suggests that OCGs in Mexico City maintained similar activity levels during the pandemic.

Finally, on the basis of content analysis of legal acts, strategic documents, academic papers, media reporting, official documents, and interviews with police officers and representatives of civil society organizations, Djordjević and Dobovšek (2020) have investigated how COVID-19 has impacted crime and crime-fighting in the Western Balkans. The authors show that, in the first nine weeks of the spread of COVID-19, the Western Balkans experienced a small rise in the price of cannabis, ecstasy, and amphetamines, which remained available; by contrast, the supply of heroin decreased. The impact of COVID-19 on human trafficking in the area was, instead, hard to establish.

The foregoing review of the available academic studies on OC and COVID-19 indicates that empirical attention to OCGs’ provision of illegal governance has been marginal, whilst OC’s infiltration of the legal economy has not yet been investigated. Moreover, studies analyzing OCGs’ reactions to the pandemic are few, which leaves several issues open for exploration. In particular, those regarding the groups that have exploited the opportunities given by COVID-19, and the means they have used to provide illegal governance and infiltrate the legal economy. Against this background, the aim of the current study is to investigate what kind of opportunities does the COVID-19 outbreak offer to OC, and what strategies do OCGs use to exploit them.

While most criminal groups are relatively small, flexible, and engage in illegal activities for monetary gains, others seek to also exercise influence on the communities of which they are part (Paoli and Vander Beken 2014). Five organizations, often labeled ‘mafia-type OCGs’, are classic examples of the latter form of OC. They are the Russian Mafia of the 1990s, the Italian Cosa Nostra and ‘ndrangheta, the yakuza in Japan, and the Triads in China (Lo and Kwok 2017; Reuter and Paoli 2020; Varese 2014). These OCGs do not merely exploit the weaknesses of states; instead, by enacting or performing state-like activities and privatizing the provision of services, they also acquire a political component (Arias 2006; Gambetta 1993). Most powerful Mexican drug-trafficking organizations, Neapolitan Camorra, as well as structured criminal organizations in Brazil, Colombian, El Salvador, Guatemala, Nigeria, and the United Kingdom, also exert control over their territories, albeit to a lesser extent and with limited governance capability (Arias 2006; Aziani et al. 2020a; Brophy 2008; Campana and Varese 2018; Cruz 2010; Reuter and Paoli 2020).

In their study on non-state governance in Sub-Saharan Africa, Raeymaekers et al. (2008) observe that crises, although destructive, can bring opportunities and change, creating new emergent orders once the situation has stabilized. This may be the case of COVID-19 and governance-type OCGs. The medical, economic, and social disarray caused by the pandemic creates the possibility for OCGs to strengthen their local political influence. Because criminal governance relies on the relation and interaction between the (absence of the) state and OCGs, the difficulties faced by Governments in mitigating the negative impact of the pandemic may favor OCGs. Indeed, irrespectively of its providers, when governance is perceived as fair and effective, it can acquire legitimacy (Felbab-Brown 2017; Lessing and Willis 2019). In turn, greater legitimacy brings direct benefits for OCGs (e.g., greater acceptance of protection payments) and eventually supports them in their competition with legal institutions for state-making (Aziani et al. 2020b; Felbab-Brown 2017). Conversely, we expect to find at most few incidents referring to less stable criminal organizations/collaborations or inward-looking criminal groups which neither aspire to nor are capable of performing forms of illegal governance in response to the COVID-19 epidemic.

Besides governance ambitions, criminal organizations have also the need and the capacity to infiltrate the legal economy in different ways for the purposes of laundering the proceeds of their illegal activities, logistically supporting those activities, and expanding their sources of income (Levi and Soudijn 2020; Paoli and Vander Beken 2014; Savona et al. 2016; Savona and Riccardi 2015). The COVID-19 pandemic provides OCGs with new opportunities to expand their infiltration. Indeed, the crisis has exposed numerous businesses to a severe shortage of capital. In the absence of sufficient and ready-to-obtain public subsidies, these businesses may be at greater risk of criminal infiltration (Stephany et al. 2020). More fragile economies are those where we expect OC infiltration to be higher because less solid companies and businesses operating in the grey economy have greater difficulties in obtaining loans from legitimate financial institutions. The banking system may not be able and willing to support struggling businesses because of increased systemic risk as well as their specific risk of loan default. On the other hand, OCGs can compensate for the higher risks because of the high liquidity at their disposal, often lent with usurious interest rates, and they can be more effective than legitimate financial institutions in debt recovery by trespassing the boundaries of legality (Lavezzi 2014; Scaglione 2014).

Opportunities have arisen not only in the private sector. By relying on corruption practices and political connections, OC may indeed infiltrate public procurement in order to illicitly obtain public funds (Caneppele et al. 2009; Pinotti 2015). Specifically, observation of past socio-economic crises shows that a large number of OCGs have been able to appropriate recovery funds, stimulus packages, and other public subsidies (Fukushima 1995; Monzini 1999). For example, this has frequently been the case of earthquakes in Southern Italy (e.g., those in Belice in 1968, Irpinia in 1980 or Abruzzo in 2009). On those occasions, Cosa Nostra, the Camorra and other OCGs took advantage of reconstruction works to obtain public procurements and subsidies, and to strengthen their grip on local businesses and markets (Monzini 1999; Parrinello 2013; Riccardi et al. 2016). This is of importance because already in the first month of the pandemic, the distress caused by the spread of the virus and by the social distancing measures induced the governments of several countries to plan unprecedented injections of liquidity into their economies (Rizwan et al. 2020). Two are the main contributing factors to the risk of embezzlement: the sheer amount of disbursements and the need for rapid action. The latter, in particular, may result in relaxed scrutiny on the use of funds. Urgency, in fact, may hinder the ability of governments to devise and activate proper public procurement policies, thus increasing the risk of criminal misappropriations.

In accordance with the previous literature on OC, we expect to find evidence of the ability of most structured groups to misappropriate public funds on a large scale by taking advantage of the massive injection of liquidity in the global economic system induced by COVID-19. On the other hand, we expect to find little to no evidence indicating that unstructured groups and criminal enterprises are able to infiltrate the legal economy and misappropriate of public funds. Indeed, as observed by von Lampe and Blokland (2020) with respect to criminal outlaw motorcycle clubs, unlike governance-type OCGs, less structured criminal groups have limited capacities to misappropriate of public funds due to their lack of connections in the upper-world.

## Methodology

The methodology implemented in our analysis was intended to find exemplificative cases of OCGs reaction to the COVID-19 situation that would serve to reconstruct how some groups have adapted to the new reality with respect to illegal governance, infiltration of the legal economy, and illicit appropriation of public funds. Data were first identified and collected through a systematic assessment of relevant information from two main sources: (1) articles by the media and (2) reports by international organizations. Our assessment of pertinent cases and events published by the media relied on international, national, and local newspapers and reports stored in the largest available digital repositories and personalized news aggregators, including the Lexis Nexis® Metabase platform and Google News. We also consulted media outlets and investigative journalists’ sources, such as the Organised Crime and Corruption Reporting Project (OCCRP) and InSight Crime. Analysis of official reports was based on public releases made by relevant organizations, including INTERPOL, Federal Agency for Medicines and Health Products (FAMHP), and Guardia di Finanza (Italy).

The data-gathering process began with the automatic identification and extraction of media articles and official releases using different sets of keywords and queries relating to the topic of OC and COVID-19 over the eight-month period January-August 2020. While media and international organizations have published many articles and reports during this time, most of them are of a speculative nature or are general commentaries, hence do not report tangible evidence. Consequently, we had to filter out only those instances where actual cases were addressed. In order to collect as much information as possible and to avoid double counting, we further triangulated information corroborating the details of an article with information from other sources referring to the same event. Then, a manual recognition of factual evidence was performed, along with classification of the identified information into the various categories of criminal behavior examined in this study (i.e., provision of illegal governance, infiltration of the legal economy, misappropriation of public funds). A criminal event was not always classified in a single class since an event may be related to several of the criminal behaviors mentioned above. For instance, infiltration of the legal economy may occur simultaneously with the misappropriation of public funds or with the involvement of illegal governance activities.

**Fig. 1:**
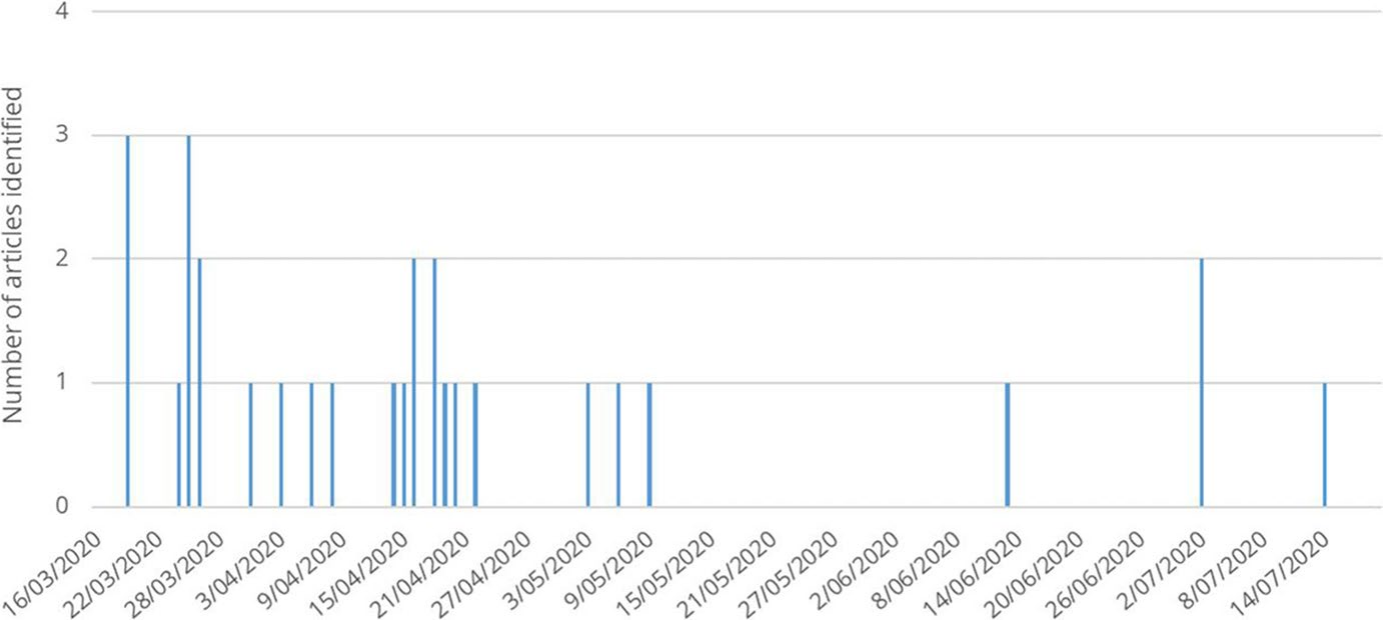
Temporal distribution of identified cases

As result, we were able to identify 29 distinctive instances of exploitation of the COVID-19 crisis by OCGs through illegal governance and/or infiltration of the legal economy. The news items and official reports analyzed were articles in English (14), Spanish (9), Italian (3), and Portuguese (3), which were retrieved from 21 sources scanned over 85,000 sources worldwide.^1^ The identified cases made reference to the strategies implemented by different criminal groups, including the distribution of products (medical products, sanitary items, food, and essential products), imposition of movement restrictions (on vehicles, local residents, and tourists), price control of primary goods, closure of businesses, trafficking of medical and sanitary products, and misappropriation of public funds (more details in Table 1). The events did not distribute evenly over time since we observed more cases reported in the two-month period from the end of March to the beginning of May (Fig. 1).

**Table 1:** Source, country of source, source language, number of identified events, and notes on the cases

*Source*	*Language*	*Country of source*	*# News articles*	*Notes on events*
*CNN*	English	US	1	• Imposition of curfews and social distancing, and distribution of food, medicine, and sanitary products in favelas in Rio de Janeiro, **Brazil**
*EconomiaHoy*	Spanish	Mexico	1	• Distribution of food by the Gulf Cartel in Ciudad Victoria, **Mexico**
*El Comercio*	Spanish	Ecuador	1	• Distribution of food and sanitary items by Joaquín Guzmán’s relatives in Guadalajara, **Mexico**
*El Faro*	Spanish	El Salvador	1	• Imposition of curfews by gangs connected to Barrio 18, Revolucionarios, and MS-13 in San Salvador, **El Salvador**
*El Nacional*	Spanish	Venezuela	1	• Compliance with hygiene and social quarantine protocols by the Tupamaros militia in Caracas, **Venezuela**
*El Periodico*	Spanish	Guatemala	1	• Gangs interrupted their extortions in **Guatemala**
*FAMHP*	English	Belgium	1	• Seizure of counterfeit and other illegal medicinal products in **Belgium**
*Foreign Policy*	English	US	1	• Provision of basic health care services and distribution of health information by the Taliban, **Afghanistan**
*G1 Globo*	Portuguese	Brazil	1	• Control of prices of primary goods to impede speculations in Rio de Janeiro, **Brazil**
*Guardia di Finanza*	Italian	Italy	1	• Embezzlement of COVID-19 emergency funds by the 'ndrangheta, **Italy**
*Il Mattino*	Italian	Italy	1	• Distribution of food and primary goods by members of Camorra in Naples, **Italy**
*INTERPOL*	English	International	2	• Global Operation Pangea III on counterfeit medical products in **Europe** • International fraud scheme on COVID-19 emergency funds
*La Opinión*	Spanish	US	3	• Distribution of food parcels and primary goods by gang members in several cities in **Mexico**
*La Repubblica*	Italian	Italy	1	• Distribution of food and primary goods by members of Cosa Nostra in Sicilia, **Italy**
*OCCRP*	English	International	4	• Distribution of counterfeit sanitary items in **Lithuania** • Distribution of counterfeit sanitary items in **Romania** • Embezzlement on COVID-19 procurement by **Slovenian** OCG • Distribution of sanitary items by Yakuza members in **Japan**
*Terra*	Portuguese	Brazil	1	• Prohibition on tourists entering the favelas by gangs in Rio de Janeiro, **Brazil**
*The Fifty-Four News*	English	International	1	• Distribution of food parcels by gangs in Cape Town, **South Africa**
*The Guardian*	English	UK	3	• Imposition of curfews and distribution of sanitary products in favelas in Rio de Janeiro, **Brazil** • Distribution of unusable sanitary items in the **UK** • Distribution of food parcels and primary goods by gang members in several cities in **Mexico**
*Times Live*	English	South Africa	1	• Distribution of food parcels and primary goods by gangs in Cape Town, **South Africa**
*TuBarco*	Spanish	Colombia	1	• Imposition of quarantine measures by dissidents formerly members of the FARC in **Colombia**
*UOL*	Portuguese	Brazil	1	• Forced closure of shops and imposition of curfews in several favelas in Rio de Janeiro, **Brazil**

### Limitations

The main limitation of the methodology employed is associated with the non-random nature of the sample of identified OC activities. It is well acknowledged that news media decision makers often rely on their own criteria when deciding whether or not to publish a story, as well as the narrative behind it (Chermak 1995). Moreover, media attention to OCGs may differ among countries and regions because criminal activities are perceived and reported differently in different contexts. As such, strategies of the medias may introduce a bias towards more sensationalist and serious crimes, perhaps committed by notorious mafia-type organizations, as editorial staffs might consider them more newsworthy than less serious offences, perhaps committed by little known criminal enterprises. Related to this, it may be the case that certain episodes not reported so far will be exposed in the future, depending on the ability of law enforcement agencies to detect them. While there is anecdotal proof suggesting that, for instance, the illegal acquisition of recovery funds and public subsidies may have increased in some countries (see e.g., DIA 2021, in Italy), there is no strong evidence to determine whether or not OCGs are able to infiltrate or to increase their governance during this period of crisis. Nevertheless, this is not something new in the study of OCGs.

Many authors have stressed the great difficulty of empirically assess and measure OC, mainly because of its ill-definition (Kleemans 2014), and the endogenous error of crime reporting (Pinotti 2020). The first refers to the vast variety of OC definitions as some researchers concentrate on long-establish organizations, some on complex and transnational groups, and others on local illegal markets. The second point relates to the fact that enforcement and official data will rarely reflect a comprehensive picture of OC activities, even in jurisdictions where enforcement and mitigating measures are effective—i.e., a low level of crime related to OC could be a good sign indicating a low level of criminal activity, or it could be a bad sign indicating a low rate of detection.

However, the aim of our analysis was not to assess the extent to which both illegal governance and infiltration in the legal economy have increased or changed; but, instead, to find empirical evidence on whether these two dynamics have occurred and if so, how. Therefore, we find value in the use of media sources for the identification of relevant cases as they provide evidence on OC activities that occur in many different places and that are often not reported to and by authorities. However, we avoided making claims on criminal patterns or trends, as they extend beyond what we can support with the collected data, and instead concentrated on the qualitative assessment of the identified articles.

## Results

### Governance

The strict lockdowns imposed by governments worldwide have sought to control and manage the health crisis. These measures have resulted in an economic downturn which has imposed stress on individuals and businesses alike (Nicola et al. 2020). Moreover, in numerous countries, the socio-economic instability has exposed two weaknesses of governing authorities: their slow, and often inadequate, economic assistance; and their difficulties in enforcing widespread lockdowns (Bonacini et al. 2021; Campedelli and D’Orsogna 2021). The review of cases suggests that numerous OCGs have attempted to exploit these weaknesses to obtain, maintain or increase their social legitimacy. Some OCGs, indeed, have engaged in multiple actions aimed at building consensus, strengthening their control over specific territories, and consolidating their bonds with local people. Overall, from the analysis of the identified cases, the means with which OCGs have tried to exert and expand their governance role during the pandemic can be classified into two broad categories. First, these groups have provided sustenance and facilitated access to resources that people need—e.g., food parcels, sanitary products, toilet paper, tissues. Second, they have imposed and enforced lockdown measures to halt the spread of the virus.

The provision of different arrangements of economic relief emerged as the most frequently reported governance strategy adopted by OCGs during the first months of the pandemic. The cases by the media account for different tactics, such as the distribution of groceries, the delivery of sanitary products, but also price controls and outright cash handouts. Food and primary goods have been distributed by Cosa Nostra and Camorra affiliates in the Italian cities of Palermo and Naples, sometimes exploiting fake charity organizations set up specifically for the purpose (Crimaldi 2020; Palazzolo 2020). Members of rival gangs in South Africa’s capital Cape Town have made truce and agreed on laying down their weapons to distribute food parcels and primary goods (Kennedy 2020). Similarly, members of numerous drug trafficking organizations in the Mexican states of Jalisco, San Luis Potosí, Veracruz, Michoacán, Tamaulipas, Guerrero, and the State of Mexico supplied bundles containing food and sanitizers to the states and cities under their influence (Agencia EFE 2020; Economìa Hoy 2020; Hyman 2020; La Opinión 2020a, b, c).

A second type of handout has consisted in the distribution of sanitary equipment like face masks, hand sanitizer and soap, as done by gang members affiliated to the Comando Vermelho operating in the favelas of western Rio de Janeiro and by yakuza members in Japan (Barretto Briso and Phillips 2020; Cerantola 2020; Ríos and Sapalú 2020). Some OCGs in Latin America have provided not only in-kind contributions; gang members of Barrio 18 in Guatemala City have suspended extortion payments, while gangs in Brazil have made outright cash handouts and prevented speculations, halting increases in the prices of basic necessities (Leitão and Martins 2020; Ríos and Sapalú 2020; Walsh et al. 2020).

OCG governance activities have also included the imposition and the enforcement of social distancing measures, and the performance of other policing activities in some cases by threatening residents with reprisals and violence. Several Brazilian gangs, some of them belonging to the aforementioned Comando Vermelho, have prohibited tourists from entering the favelas in Rio de Janeiro while also imposing the closure of shops (Barsetti 2020; Blois 2020). In the Morro dos Prazeres, citizens have been ordered to avoid circulating in groups larger than two. In Rocinha traffickers have imposed a curfew, while in Complexo da Maré they have ordered churches to reduce their operating hours (Barretto Briso and Phillips 2020). Similarly, gangs in Venezuela and El Salvador have enforced government-mandated lockdowns and restricted public gatherings in the territories under their control (El Nacional 2020; Martínez et al. 2020). While gang members in Brazil have enforced social distancing measures in response to the government’s lack of action, members of the Colectivo Tupamaro in Caracas, and of Mara Salvatrucha-13 and Barrio 18 in San Salvador, have enforced government-mandated lockdowns in and nearby controlled territories (Barretto Briso and Phillips 2020; El Nacional 2020; Martínez et al. 2020).

Not all OCGs, however, have been reported to be involved in illicit governance activities. We found no evidence, for instance, with respect to street gangs or motorcycle gangs in the United States and Australia, or trafficking networks in any region. Instead, we found cases of other criminal insurgency non-state entities that did engage in governance activities. Fighters from the dissident 29^th^ Front of the ex-FARC in Colombia, the Taliban in Afghanistan, and the Hayat Tahrir al-Sham in Syria have all engaged in policing activities, enforcing quarantine measures and restricting public gatherings (Sieff et al. 2020; TuBarco 2020). Some, like the Taliban and Hayat Tahrir al-Sham, have gone as far as substituting the government in providing basic health care services to locals by sending health teams to remote communities, distributing health information, and performing door-to-door temperature checks (Jackson 2020; Sieff et al. 2020).

Many of the identified governance activities performed by OCGs and other illegal organizations have involved blatant acts designed to ensure that the locals knew who the ‘benefactors’ were. Although OCGs mainly seek approval and legitimacy in the territories under their control, some of these activities have been directed towards a larger audience, possibly in an effort to extend their reputational reach. Social media have played a key role in this process, with several groups advertising their actions through videos and pictures. Mexican drug-trafficking syndicates, indeed, have posted images of the distribution of food parcels displaying their logo (Economìa Hoy 2020; Ernst 2020; Jorgic 2020; La Opinión 2020a). For instance, the Cártel del Golfo printed the ‘CDG’ logo on the packages it delivered or sealed them with stickers that read: “Gulf Cartel, in support of Ciudad Victoria, Mr. 46, Vaquero” being Mr. 46 the pseudonym of the leader of the Cártel del Golfo group that operates in Matamoros (Sieff et al. 2020; Torres 2020). The Cártel de Sinaloa, the Cártel de Jalisco Nueva Generación, and Los Viagras posted online videos in which their members distributed bags of groceries (Keyser 2020; Torres 2020).^2^ In a similar fashion, the yakuza have secured indirect exposure through news outlets by offering to disinfect the Diamond Princess,^3^ a cruise ship at the center of both the national and international debate concerning the pandemic (Cerantola 2020).

### Infiltration of the economy

Analysis of the cases found in the media and official reports shows that the COVID-19 emergency has provided new opportunities for OCGs to infiltrate the legal economy. The available evidence indicates that infiltration has occurred in the running of businesses involved in the sale of health products, the misappropriation of medical funds, and the embezzlement of economic stimulus packages. The economic sector identified as being most vulnerable to and exploited by criminal infiltration during the first phase of the COVID-19 outbreak was wholesale trade in medical and pharmaceutical products. In this case, OCGs were able to benefit along the entire ‘spectrum’ of possible activities, ranging from illicit ones, like the manufacturing and marketing of counterfeit product, to licit ones, like the acquisition and establishment of fully legitimate firms (e.g. ones active in the wholesale of medical devices, masks, pharmaceuticals), or their use as covers for illicit trafficking (Smith 1980). Operation *Pangea XIII* has accounted for this reality, which has been coordinated by Interpol and involved police, customs and health regulatory authorities from 90 countries (INTERPOL 2020a), resulting in 121 arrests worldwide and the seizure of many dangerous pharmaceuticals and medical products. Other cases of large-scale distribution of counterfeit and defective pharmaceutical products have been reported by law enforcement agencies in several European countries, such as Romania (OCCRP 2020a), the United Kingdom (Brignall 2020), Belgium (FAMHP 2020) and Lithuania (OCCRP 2020b), with the latter exhibiting proven connections to Romanian organized crime. The fact that some seized pharmaceutical products were counterfeit suggests that there is an ongoing hybridization of infiltration in the international distribution and actual trafficking of illicit goods within the medical environment.

Our analysis also allowed us to identify cases of misuse of public funds intended both to support medical needs and to stimulate the economic system. These included cases of corruption in the procurement process whereby funds intended for protective equipment for COVID-19 were misappropriated and transferred to a well-known OC member in Slovenia (OCCRP 2020c). In addition, we have found a case of a massive fraud scheme coupled with money laundering concerning the supply of 10 million face masks worth €15 million in which German health authorities were scammed by criminal groups who orchestrated a complex chain of referrals between suppliers in Spain, Ireland and the Netherlands (INTERPOL 2020b). Finally, the analyses highlighted a case of embezzlement of Italian COVID-19 funds in which economic relief packages valued at €45,000 were awarded to a ‘ndrangheta member through a complex scheme of tax fraud, false invoices and fake names within the steel trade sector (Guardia di Finanza 2020). As said, it is possible that the misappropriation of public funds by OCGs has increased starting after the studied period, since most recovery-plans were authorized and officially implemented only in the last months of 2020.

The media debate on the possibility of OC infiltration in sectors weakened by the pandemic has been vast (Agenpress 2020; Dellasega and Vorrath 2020). The rapid surge in demand in specific sectors is what made them particularly attractive for speculators. Sectors such as the health care, logistics and e-commerce all boomed due to the health crises and lockdown measures; the food retail trade and cleaning services grew because of disinfestation requirements introduced in most countries; waste management and funeral services expanded due to the increased mortality. Other sectors, on the other hand, became vulnerable due to financial distress. These include tourism, hospitality, transportation, beauty salons, arts and entertainment, and recreation. Nevertheless, we found no official cases of infiltration to support these speculations except for the aforementioned wholesale trade of medical and pharmaceutical products. Again, evidence may emerge in the second phase of the pandemic when ongoing and future investigations may bring forth significant evidence.

## Discussion

The analysis performed indicates that several criminal syndicates exploited the spread of COVID-19 in the first phase of the pandemic to show themselves as providers of governance and to benefit either in terms of *power* or *profit* from their infiltration of the legal economy. There is evidence with respect to five dynamics: the provision of goods in high demand; the imposition of social-distancing measures; the deferral of the collection of protection payments from local businesses; the infiltration of industries, which the crisis made more lucrative; the misappropriation of funds intended to mitigate the impact of social distancing on economies. In the first eight months after the spread of SARS-CoV-2, illegal governance activities were more commonly reported by the news than activities related to infiltration of legal economy and misappropriation of public funds. On the one hand, this result might emerge because governance activities may take place prior to episodes of infiltration in the legal economy. Enforcing social distancing or providing food to communities experiencing hardship requires little planning or organization. On the other hand, illegal governance measures are more public and explicit than attempts to infiltrate the legal economy, as the Mexican cases exemplify. As such, they are also more likely to be reported by media and institutional reports.

Not all forms of OCGs have been found to engage in these actions of governance. In line with the theoretical expectation that the differences in activities performed by OCGs depend on their nature, our empirical results provide evidence on some mafia-type OCGs exploiting COVID-19 to affirm themselves as providers of illegal governance. In this regard, Cosa Nostra and Camorra groups in Sicily and Campania or Yamaguchi-Gumi in Yokohama supply the population with sanitizers or food, as well as proposing themselves as providers of other forms of economic support. Members of the Comando Vermelho have imposed price controls on goods in high demand because of the pandemic; at the same time, they have imposed and enforced stay-at-home restrictions on the people living in some favelas under their influence. Even if the popular legitimacy of the Comando Vermelho is often ephemeral, this behavior aligns with previous cases of leaders of gangs belonging to the organization who have spent considerable sums of money on benevolent actions, or provided security governance to the favelas where they are based (Reuter and Paoli 2020). Conversely, although authors such as Lessing and Willis (2019) show that the Primeiro Comando da Capital originating in Sao Paulo provides illegal governance, the group was not explicitly mentioned in any specific case. This difference, however, may be a byproduct of the bias in the methodology presented in earlier sections Indeed, sources often referred to ‘gangs’ and ‘gang members’ in general without providing enough details on the matter. Hence, affiliations were not always explicit and clear. Although the evidence presented in this papers is exploratory and criminal groups are heterogeneous—e.g., South African gangs and Cosa Nostra—, the results indicate that support for people in need and the imposition of common rules during the pandemic have been performed mainly by OCGs with pre-existing political power and/or connection with the local population. Whether this is driven by the desire to acquire greater economic and organizational capabilities, or to increase the organization’s power within the community remains to be investigated by further studies.

By contrast, no evidence was found on illicit governance enforced by criminal groups not strongly rooted in their territory before the pandemic. No reports, indeed, account for the involvement of youth gangs, outlaw motorcycle gangs, and trafficking networks in forms of illegal governance in the aftermath of the pandemic. This may be due to the fact that youth gangs, for instance, are mainly focused on their members and are not used to reaching outside their groups (Lafontaine et al. 2009; Soliz and Rittenour 2012). Consequently, a change in the social landscape does not drive them towards a greater role in the in the community and political environment, nor pushes them to become multifunctional in reaction to the crisis. These findings align with those by Kim and Phillips (2021), who observed a significant increase in gang-related gun violence in Buffalo, New York, following the COVID-19 outbreak, and further interpreted it in light of increased strain on gang members. Kim and Phillips (2021) argue that the pandemic has led to an unprecedented increase in the unemployment rate, especially for poor individuals in the inner city, which in turn has led to an increase in gun violence. This dynamic clearly contrasts with the one of powerful groups organizing actions to support people in need in a showy way. A similar line of reasoning may apply to outlaw motorcycle gangs and trafficking networks, as these organizations already lacked the incentives and means to provide governance before of the turmoil caused by COVID-19 (von Lampe and Blokland 2020).

It is plausible that the provision of goods in high demand is primarily intended to strengthen bonds with the local population while discrediting legitimate institutions, highlighting the inability on national and local government to help the community. The endeavor by some OCGs to give visibility to their donations supports this interpretation; OCGs seek to maximize their audiences, thus obtaining higher returns on their investments. Likewise, by enforcing lockdowns and stay-at-home orders, OCGs try to propose themselves as socially legitimated governing institutions. All these actions are functional to obtaining a bottom-up legitimization. The only exception found in the collected sample is the attempt by the yakuza to be recognized as a legitimate interlocutor by the Japanese government by offering to sanitize the cruise ship blocked in Yokohama. Nevertheless, the yakuza did not achieve this form of top-down legitimacy because the Japanese government refused its offer.

Additionally, expansion of governance in this turbulent period may facilitate infiltration of the legal economy. In fact, the two dynamics are not always clearly separated since one can accompany and serve the other (Savona et al. 2016; Riccardi 2014; Campana and Varese 2018). Infiltration of firms and the legal economy at large, misappropriation and misuse of public funds, and criminal governance are interconnected. For mafia-type OCGs, a greater consensus by the local community may turn into political support and facilitate control and influence over the local government and, in turn, the manipulation of public tenders. In other cases, the link may be simpler and more direct. For instance, the provision of loans at very low interest, like the ones provided by Camorra or the Unión Tepito Cartel, may directly create a bond between the business owner and the criminal group which is conductive to the later acquisition by the OC group of the company’s assets or share capital.

Among the sectors that have been reported to be most vulnerable to criminal infiltration during the first phase of the COVID-19 outbreak, the wholesale distribution of medical pharmaceutical products emerged as the main one. This is likely due to the sudden increase in demand for these products, which made their trade particularly profitable and possibly subject to little scrutiny by public authorities. The activity of some OCGs in this sector involves the combined manufacturing and trafficking of counterfeit or defective medical devices and pharmaceuticals, and the establishment of new legal firms (often used as covers for illicit activities). Whilst the identification of targeted sectors is important for safeguarding the integrity of legitimate economies, it is essential to identify cases of firms that may be more prone to infiltration. If any becomes available, future studies may want to focus on the incident level, rather than the structural level. Moreover, whereas we have observed the short-term effects, the medium- and long-term ones are also relevant and possibly different. As said, recent anecdotal evidence suggests that the misuse of public funds by organized crime has expanded with respect to the first phase of the pandemic, also because recovery plans have only recently entered into force.

Close attention has been paid to the possibility of OC infiltration in vulnerable sectors. Experts on the topic expect that, with the prolongation of the COVID-19-induced crisis, three main areas will be challenged by infiltration risks: (i) sectors made vulnerable by the crisis and hence with financial difficulties, such as tourism, hospitality and transport, (ii) sectors made attractive by the crisis, such as health care, cleaning, food and logistics, and, finally, (iii) public funds and potential victims of fraud and money laundering (Dellasega and Vorrath 2020; GITOC 2020b). Although we have identified some cases of OC infiltration in the healthcare sector and misappropriation of COVID-19 funds, we were unable to bring robust evidence in support of OCGs actual capacity to misappropriate public funds intended to support the recovery of national economies. This may be due to the long-term nature of the phenomena considered and to the difficulty encountered by enforcement institutions in gathering enough evidence to carry out comprehensive investigations and operations.

## Conclusions

This study has explored cases from the media and official releases on the immediate adaptation of OCGs to the spread of COVID-19 and to the implementation of containment and economic-relief policies adopted by governments around the world in the first phase of the pandemic. Among the possible changes in OCGs activities, we have focused on the provision of illegal governance and on the infiltration of the legal economy, since these are dynamics particularly threatening for the functioning of societies and legitimate economic systems when crises are in place. The analysis of illustrative cases shows evidence on the fact that the COVID-19 emergency has affected the behavior of some OCGs by providing them with new opportunities to gain legitimacy and support in the community within which they operate, as well as to expand and diversify their investments. Around the world, governance-type OCGs have provided relief packages, enforced social distancing, and imposed price controls; we have collected no evidence of trafficking networks doing the same. Direct evidence of OCGs infiltrating the legal economy is less abundant; nonetheless, it exists and it may be expected to become more visible in the medium and long term. In the first eight months after the spread of COVID-19, there occurred episodes of OCGs trying to exploit the growing demand for medical and pharmaceutical products, and to embezzling public funds. These findings are necessarily preliminary and exploratory, and they will have to be triangulated and verified once official statistics and additional information become available. Nevertheless, they furnish insights into dynamics to be considered when devising anti-crime and economic-relief policies.

During the pandemic, OCG governance activities have somewhat aligned with government interests, highlighting the complexity of the relation between criminal governance and legitimate state governance. Rather than necessarily being in immediate opposition to one another, the two can sometimes align, posing additional challenges for legitimate governing authorities. For instance, a crackdown on OCGs enforcing government-mandated lockdowns is difficult to justify in the eyes of citizens, who may deem those actions inconsistent, thus further increasing the distance between them and the state. In the long term, however, any legitimization of OCGs threatens the health of societies, solidifying the position and status of OCGs in the community. It highlights the failures of governments and diminishes their legitimacy, ultimately making it easier for OCGs to continue their activities in the future and to swell their ranks.

Illicit governance, infiltration of the legal economy, and the embezzlement of public funds should not be compartmentalized and treated as three unrelated dynamics. Policy-makers, in fact, should design policies which take account of the interconnectedness among these elements, carefully evaluating the trade-offs, and finding the balance among them. First, activities of governance and infiltration of the legal economy are sometimes similar in form. Because the pandemic has led to both individuals and businesses being strapped for cash, some OCGs have had the opportunity to provide money and resources both to engender loyalty and to acquire shares in legal businesses. Second, government-issued stimulus packages entail a trade-off between the risk of embezzlement, on the one hand, and the risk of infiltration and strengthening illicit governance on the other. Sizeable liquidity injections, fiscal stimulus, grants, and other funds intended to accelerate economic recovery carry an inherent risk of embezzlement. This risk is further exacerbated by the weakened institutions, the need for swift interventions and—possibly—relaxed scrutiny and insufficient safeguards.

Adequate subsidies, however, are vital for preventing a long and disastrous recession. Too small and prudent interventions may leave businesses and people on the brink of bankruptcy, opening the way for illicit sources of funding. Weakened balance sheets and grim prospects concerning future earnings, indeed, are fertile ground for OCGs’ infiltration. Unlike the 2008 financial crisis, banks are not in distress; but the inability of borrowers to provide adequate collateral and the banks' unwillingness to lend money in a high-risk environment may limit the injection of private capitals in the market. OCGs, on the other hand, have illicit ways to coerce borrowers into paying back loans or giving up collateral. To conclude, governments are faced with the difficult task of striking the right balance between large and swift liquidity injections, which may be misappropriated and misused by OCGs, and insufficient aid, which may foster infiltration and strengthen the social legitimacy of criminal groups.

## Footnotes

1 A language bias may have been present because we employed a limited number of languages; for example, no news items in Japanese, Chinese, Arabic, or other Asian languages were scanned and retrieved.

2 The news article by Jorgic (2020), Keyser (2020), Torres (2020) present the same events as described in other news articles reported in Table 1; they are used as a reference because they provide a more detailed description of the use of social media and other promotional activities by Mexican drug-trafficking organizations. However, these news articles were not added to Table 1 in order to avoid double-counting issues and maintain an accurate account of the events.

3 The Diamond Princess was a cruise ship which docked and quarantined in the port of Yokohama on 4 February 2020. Between 20 January and 1 March, 712 passengers tested positive for the virus, resulting in 14 casualties.
